# Effectiveness of enhanced cognitive behavior therapy for bulimia nervosa in Japan: a randomized controlled trial protocol

**DOI:** 10.1186/s13030-020-0174-z

**Published:** 2020-02-24

**Authors:** Chisato Ohara, Atsushi Sekiguchi, Shu Takakura, Yuka Endo, Naho Tamura, Hiroe Kikuchi, Kazushi Maruo, Norio Sugawara, Kenji Hatano, Hitomi Kawanishi, Misako Funaba, Ayako Sugawara, Nobuhiro Nohara, Keisuke Kawai, Shin Fukudo, Nobuyuki Sudo, Zafra Cooper, Kazuhiro Yoshiuchi, Tetsuya Ando

**Affiliations:** 1 Department of Behavioral Medicine, National Institute of Mental Health, National Center of Neurology and Psychiatry, 4-1-1 Ogawa-Higashi, Kodaira, Tokyo, 187-8553 Japan; 2 Department of Psychosomatic Medicine, Kyushu University Hospital, Fukuoka, Japan; 3 Department of Psychosomatic Medicine, Tohoku University Hospital, Sendai, Japan; 4 Department of Psychosomatic Medicine, Kohnodai Hospital, National Center for Global Health and Medicine, Ichikawa, Japan; 5 Department of Psychosomatic Medicine, Center Hospital, National Center for Global Health and Medicine, Tokyo, Japan; 6 Department of Biostatistics, Faculty of Medicine, University of Tsukuba, Tsukuba, Japan; 7 Department of Psychiatry, Dokkyo Medical University School of Medicine, Mibu, Japan; 8 Department of Clinical Epidemiology, Translational Medical Center, National Center of Neurology and Psychiatry, Kodaira, Japan; 9 Department of Stress Sciences and Psychosomatic Medicine, Graduate School of Medicine, The University of Tokyo, Tokyo, Japan; 10 Graduate School of Medicine, Department of Behavioral Medicine, Tohoku University, Sendai, Japan; 11 Department of Psychiatry, Yale School of Medicine, Yale University, New Haven, CT USA

**Keywords:** Eating disorder, Bulimia nervosa, Cognitive behavior therapy, Randomized controlled trial

## Abstract

**Background:**

The effectiveness of psychotherapeutic interventions for eating disorders (EDs) is widely studied in Europe, North America, and Australia/New Zealand. However, few controlled studies and no randomized controlled trials (RCTs) have been conducted in Japan despite the relatively high prevalence of EDs in the Japanese population. The aim of this study is to evaluate the effect of enhanced cognitive behavior therapy (CBT-E), an evidence-supported ED-focused form of cognitive behavior therapy (CBT), for the treatment of bulimia nervosa (BN) in Japan.

**Methods/design:**

This multicenter RCT will compare CBT-E with treatment as usual (TAU), which is widely used in Japan. A group of 140 adult outpatients with a Diagnostic and Statistical Manual of Mental Disorders Fifth Edition (DSM-5) diagnosis of BN, ≥18 years of age, a body mass index (BMI) > 17.5 and < 40 kg/m^2^ will be randomly assigned to CBT-E or TAU. Participants will be stratified by intervention site and BN severity. CBT-E participants will receive 20 sessions of focused form CBT-E for 20 weeks. Those in the TAU group will receive routine treatment provided by specialists. Assessment will be performed in a blinded manner prior to the start of treatment, after 6 weeks of treatment, at the end of treatment (20 weeks), and at follow-up at 40 and 80 weeks after the start of treatment. The primary outcome is the remission of BN, defined by the absence, in the previous 4 weeks, of symptoms required to meet the DSM-5 criteria for a diagnosis of BN. Secondary outcomes include the levels of ED psychopathology and impairment due to the ED, anxiety, depression, family function, and satisfaction with treatment.

**Discussion:**

This will be the first RCT conducted in Japan to compare CBT-E and TAU for the treatment of BN. If CBT-E is found to be more effective than TAU, then the evidence would support its wider use for patients with BN in Japan. Because it is possible to train therapists who do not possess extensive specialist experience, wider use is also likely to be practically feasible. In addition, demonstrating the effectiveness of CBT-E in Japan would demonstrate that it could be successfully extended to additional world cultures and regions.

**Trial registration:**

UMIN, UMIN000031625. Registered 7 Mar 2018.

## Background

Bulimia nervosa (BN), anorexia nervosa (AN), and binge-eating disorder are the three specific eating disorders (EDs) included in the Diagnostic and Statistical Manual of Mental Disorders, Fifth Edition (DSM-5). A diagnosis of BN requires recurrent episodes of binge eating, recurrent inappropriate compensatory behaviors designed to prevent weight gain (e.g., self-induced vomiting, misuse of laxatives and diuretics, and over-exercising), and self-evaluation that is unduly influenced by body shape and weight [1]. BN is associated with psychological and social impairment [2], elevated risk of medical complications, and mortality [3], and psychiatric comorbidities such as depression, anxiety and personality disorders [4, 5]. Elevated risk of death from all causes and suicide, with a standardized mortality ratio of approximately 2, has been reported [6]. BN tends to run a chronic course, particularly when untreated. Severe eating pathologies have been reported in up to 50% of those diagnosed with BN at 5-year follow-up, with most not having received treatment [2, 7]. The lifetime prevalence of BN in women in Europe and the United States has been estimated as 0.5–3% [2, 8–11]. The point prevalence of BN in young women in Japan has been estimated as 1.9–2.9% [12]. Nakai et al. reported that the prevalence of BN significantly increased between 1982 and 2002 with a point prevalence of 2.3% in female students aged 18–23 years in 2002 [13]. BN thus appears to be relatively common in Japan.

Treatment as usual (TAU) for BN in Japan is individualized to meet patient needs and includes: establishing a therapeutic relationship; psychoeducation; motivation to change behavior; nutritional guidance; recording eating behavior and using behavioral techniques (e.g., stimulus-control methods); supportive psychotherapy; family support; group therapy; and psycho-pharmacotherapy. Enhanced CBT (CBT-E) is rarely available in Japan despite recommendations that it is the psychological treatment of choice for ED in a number of national clinical guidelines, including the UK National Institute for Health and Care Excellence in 2004 and 2017 [14, 15], the American Psychiatric Association in 2006 [16], and the Royal Australian and New Zealand College of Psychiatrists in 2014 [17]. Although its adoption by national guidelines reflects the strength of the evidence supporting the treatment, there are currently no empirical data supporting its use with Japanese patients.

CBT was first described as a treatment for BN in the early 1980s [18], a time when the disorder was newly recognized and considered intractable. Since then, CBT theory has become increasingly concerned with the processes that maintain BN rather than with accounting for its development [19]. CBT for BN (CBT-BN) has been endorsed by the National Institute for Health and Care Excellence [14] as the leading treatment on the basis of evidence derived from numerous randomized controlled trials (RCTs). Subsequently an enhanced version of the theory and treatment (CBT-E) have been developed which is transdiagnostic in scope [20]. CBT-E and the theory on which it is based was extended and enhanced in two respects. First, it was extended to cover all EDs. EDs share the same distinctive core psychopathology, in other words, “over-evaluation of shape and weight and their control”, regardless of the ED diagnosis. This psychopathology is expressed in features characteristic to EDs, such as strict dieting, binge eating, and various inappropriate weight control behaviors. Second, CBT-BN needed to be improved because less than half the patients who received the treatment achieved full and lasting recovery. Consequently, it was extended to address four additional mechanisms that act to maintain EDs and constitute obstacles to change [21]. These are the influence of extreme perfectionism (“clinical perfectionism”), difficulty in coping with intense mood states (“mood intolerance”), unconditional and pervasive low self-esteem (“core low self-esteem”), and interpersonal problems (“interpersonal difficulties”).

CBT-E is available in 20-session versions for not-underweight patients with a BMI of ≥17.5 and in 40-session versions for low-weight patients. CBT-E is also available in two forms. The “focused form” concentrates on the core eating disorder psychopathology, whereas the “broad form” addresses one or more of the four additional mechanisms in addition to the core psychopathology. The focused form is the default and simpler form of CBT-E and is suitable for the majority of patients. The broad form is rather less effective for the majority of patients with simple psychopathology but more effective for the minority of patients with marked additional mechanisms [22].

Available evidence supports CBT-E as the first-choice treatment of BN. CBT-E was more effective in treating BN and other EDs not involving significantly low weight than a control period of waiting for treatment [22], psychoanalytic psychotherapy [23] or interpersonal psychotherapy [24]. One study showed that a new psychotherapeutic treatment for BN might be as effective as CBT-E [25]. Recent meta-analyses have provided robust evidence that CBT-E is an effective treatment for adult patients with an ED, particularly for BN, and those with other non-low weight EDs [26–28].

There have been few studies regarding the effects of psychological treatments for EDs in Japan. A single-arm pilot study provided preliminary evidence about the feasibility of guided self-help treatments based on CBT [29], but there have been no RCTs of psychological treatments that include formal protocols or treatment manuals. The lack of supporting evidence is most likely one of the reasons that ED-focused CBT has not been widely implemented in Japan.

### Research objectives and hypothesis

The aim of this RCT is to compare CBT-E with TAU in Japanese patients with BN. The hypothesis is that there will be a higher percentage of patients in remission at the end of treatment and follow-up among those receiving CBT-E than those receiving TAU.

## Methods

### Study setting and design

**Fig. 1:**
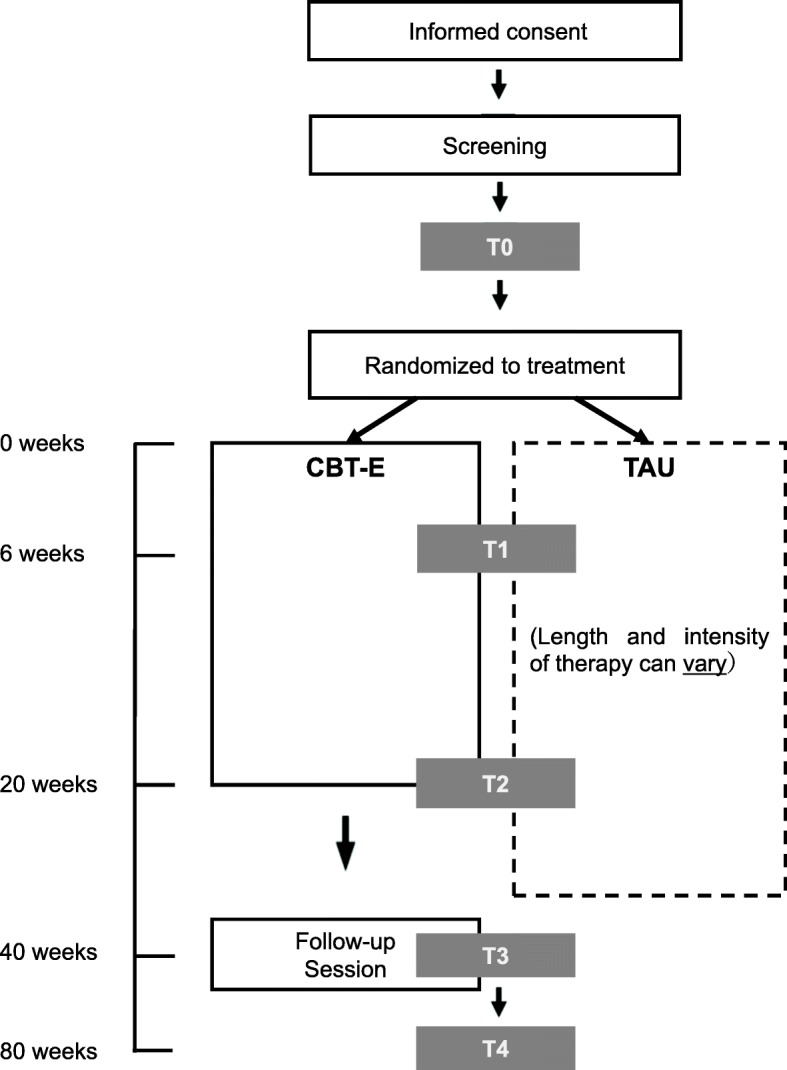
Flow of Participants through the study

This two-arm, parallel-group multicenter RCT will compare CBT-E and TAU in patients enrolled at the outpatient clinics of three university hospitals and two national hospitals in four regions of Japan. Participants will be randomized to one of the two treatments and stratified by both the study center and BN severity. TAU was chosen as the comparator because the study objective is to determine the potential benefits of CBT-E compared with a treatment that is routinely available and used in Japan [30]. Study assessments will be conducted at week 0 (before the start of treatment), week 6, week 20 (the end of treatment), and at follow-up at weeks 40 and 80. Figure 1 shows the trial design and patient flow. Clinical outcomes will be assessed by investigators blinded to the treatment allocation.

### Participants

We will recruit a group of 140 outpatients with DSM-5 BN, aged ≥18 years with a BMI of > 17.5 and a weight of < 40 kg/m^2^ from the community using advertisements. To recruit a wide range of patients, we will place no restrictions on gender or high age limits but will exclude extreme obesity, in accordance with the CBT-E guide [24] and previous studies [31].

### Inclusion criteria

Persons living in Japan, able to speak and write Japanese, and who provide voluntary written informed consent are eligible for inclusion.

### Exclusion criteria

To prevent confounding factors in our results, patients who have previously received CBT, interpersonal psychotherapy, or any similar structured psychotherapy will not be eligible. The reason for the exclusion of patients who have previously received IPT is that its effects could appear a long time after the conclusion of the therapy itself [32].

Other exclusion criteria are major psychiatric diseases such as schizophrenia, bipolar disorder, substance abuse-related disorders, or somatic diseases that could interfere with the ED treatment. Patients taking psychotropic medications other than antidepressants, anxiolytics and sleep inducers, those with intellectual disabilities, at immediate risk of suicide, not able to attend treatment sessions, or considered clinically unsuitable on safety grounds for outpatient treatment by the principal investigator and research team are not eligible. In addition, pregnant or lactating women will be excluded.

### Interventions

Interventions will be conducted at the outpatient clinics of participating hospitals. Participants in both study groups will get a medical assessment and management by doctors if necessary. Current medications will neither be changed nor will the doses be increased for 40 weeks from the start of the intervention. Antipsychotics for mental illness are prohibited except for antidepressants for depression, anxiolytics for anxiety symptoms, and sleeping pills for sleep disorders. Concurrent other structured psychosocial treatments are not permitted.

#### CBT-E

In this study, we will use the 20-session version, the focused form of CBT-E. Treatment will involve following the authorized Japanese translation of the original English language version [33]. The treatment comprises one 90-min initial assessment (session 0), followed by 19 50-min sessions for 20 weeks and a post-treatment review session.

CBT-E aims to identify the processes that maintain the patient’s psychopathology by constructing a formulation of these features and their role in maintenance and using this to guide treatment.

CBT-E consists of four stages. Stage one (sessions 0–7) is conducted with appointments twice a week. The therapist develops a collaborative relationship with the patient to involve them in CBT-E. The objective is to provide personalized education to increase the understanding of the eating problem by engaging in the real time self-monitoring at regular weighing sessions and by establishing a pattern of regular eating. Stage two comprises two sessions at weekly intervals (sessions 8 and 9) with the following aims: reviewing progress; addressing obstacles encountered; reviewing and further developing the treatment plan; and deciding whether to use the broad form of the treatment (note this is not applicable in this study because only the focused form is being investigated). Stage three consists of sessions 10–17, which are conducted once weekly. These sessions are focused on the processes that maintain the individual’s eating problem. Usually this involves addressing concerns regarding shape and eating as well as other maintaining factors such as the role of adverse events and moods. Stage four consists of sessions 18–20 and is conducted bi-weekly. The aims are to review progress; phase out particular treatment procedures and devise a plan to maintain progress; and deal with future setbacks. A final post-treatment review session will be conducted 20 weeks after the completion of treatment and is intended to review the patient’s progress and address any remaining or recurring problems. A detailed treatment guide is available [31].

#### TAU

TAU is the comparator treatment that serves as the control arm. It is the most commonly used psychosocial therapy for EDs in Japan. It includes diagnosis, assessment, establishing a therapeutic relationship, psychoeducation, nutritional guidance, recording eating behavior and behavioral change using stimulus-control methods, supportive psychotherapy, family support, and group psychotherapy. TAU is provided at the study centers as a unstructured therapy without a treatment manual and is carried out following the “Treatment Guidelines for Eating Disorders” published by the Japan Society of Eating Disorders [30]. The duration of the sessions is 15–30 min and the frequency of the sessions varies from once a week to once a month. The duration of the intervention and the total number of sessions is neither specified nor limited.

### Therapist selection and training

CBT-E therapists will be either medical doctors with specialty training in psychosomatic medicine or clinical psychologists trained by Zafra Cooper or by fully trained therapists who received training from (ZC). At least one fully trained therapist at each study center has attended a two-day CBT-E workshop given by Christopher Fairburn and 20 videoconference supervision sessions led by Zafra Cooper (ZC). Other therapists have attended CBT-E lectures in Japan followed by supervision sessions by ZC or by fully trained therapists. All CBT-E therapists will have treated at least two ED cases using CBT-E under supervision before participating in the trial. All TAU therapists will be medical doctors specialized in psychosomatic medicine and not trained in CBT-E. Both CBT-E and TAU therapists have more than 2 years of experience in treating ED. CBT-E intervention will be performed under the supervision of fully trained therapists throughout the study. CBT-E sessions will be recorded for treatment fidelity. Two sessions, one from stage one and another from stage three will be randomly selected from each CBT-E case and evaluated for fidelity. Psychologists who are familiar with the CBT-E protocol will independently use a checklist to assess treatment fidelity [33]. The content of TAU treatment will be recorded using a checklist.

### Participant recruitment

Participants will be recruited from the community by advertisements. Potential participants who give informed consent will be screened to determine eligibility. Eligible patients who are included in this study receive a pretreatment (T0) assessment and will be randomized to treatment. The study timeline and participant selection are shown in Table 1.

**Table 1:** Schedule of assessments

	Screening	T0	T1	T2	T3	T4
		Week 0	Week 6	Week 20	Week 40	Week 80
Demographic information	●					
MINI	●					
EDE		●		●	●	●
EDE-Q		●	●	●	●	●
CIA		●	●	●	●	●
BDI-II		●	●	●	●	●
STAI		●	●	●	●	●
SCL90R		●	●	●	●	●
GF-FAD		●	●	●	●	●
VAS of expectation		●	●			
VAS of satisfaction			●	●	●	●

### Outcomes

#### Primary outcome

The primary outcome is remission from DSM 5 BN at the end of treatment (T2, 20 weeks after treatment has stared). Our criteria of remission in this study comprise 1) binge-eating episodes and inappropriate compensatory behaviors occurred, on an average, less than once weekly for the previous 4 weeks, 2) self-evaluation was not unduly influenced by either body shape or weight in the previous 4 weeks (EDE score less than 4), 3) BMI > 17.5. These criteria also exclude meeting the criteria for DSM 5 AN or binge eating disorder. The remission is also assessed at follow-up at T3 (40 weeks) and T4 (80 weeks).

#### Secondary outcomes

Secondary outcomes are as follows: Change in eating disorder psychopathology and ED symptoms as measured by the EDE questionnaire (EDE-Q) and change in the severity of impairment due to the EDs as measured by the Clinical Impairment Assessment (CIA). Changes in general psychopathology including depression, anxiety and other conditions comprising a broad range of psychological problems. The effects of participant demographic information, family function, treatment expectation and satisfaction, and other variables will be considered as possible predictors or moderators of treatment.

### Measurements

#### Screening

##### Mini International Neuropsychiatric Interview (MINI)

Participants will be screened for 15 psychiatric disorders using the MINI interview, which has high diagnostic concordance rates with the ICD-10 and DSM-IV [34]. The Japanese version of the MINI has been validated [35].

#### Outcomes

##### Eating Disorder Examination (EDE)

The EDE is an interviewer-administered measure used as the gold standard for the assessment of EDs [36, 37]. It generates ED diagnoses and provides information on the frequency of ED behaviors, such as binge eating and purging. It includes four subscale scores that reflect the cognitive aspects of ED pathology and provides a global score of overall psychopathology. Weight will be measured at the interview session.

##### Eating Disorder Examination Questionnaire (EDE-Q)

The EDE-Q is a self-report questionnaire adapted from the interview-based EDE. It comprises 36 items that are scored on a 7-point scale and measures cognitive and behavioral features of EDs that occurred within the previous 28 days [38]. The total score indicates the severity of ED, with a higher score indicating more severe pathology. The EDE-Q is commonly used and has been extensively validated [37]. The reliability and validity of the Japanese version of EDE-Q has been reported [39].

##### Clinical Impairment Assessment Questionnaire (CIA)

The CIA is a self-report questionnaire designed to assess the severity of ED-associated psychosocial impairment associated with the domains of mood and self-perception, cognitive functioning, interpersonal function, and work performance within the previous 28 days [40]. It consists of 16 items that are scored on a 4-point scale and provides a global score of the severity of psychosocial impairment secondary to an ED. Higher scores indicate greater psychosocial impairment. The reliability and validity of the Japanese version have been reported [41].

##### Beck Depression Inventory (BDI)-II

This questionnaire is a self-report instrument that assesses the existence and severity of depression symptoms such as sadness, pessimism, suicidal thoughts or wishes, tiredness or fatigue, lack of energy, and lack of pleasure, among others [42]. It consists of 21 items that are scored on a 4-point scale ranging from 0 to 3. Higher scores indicate more severe depression symptoms. The BDI-II has excellent reliability and validity, and can distinguish depressed and non-depressed subjects [43]. The reliability and validity of the Japanese version have been confirmed [44].

##### State-Trait Anxiety Inventory (STAI)

This is a self-report questionnaire that includes separate measures of the anxiety state and its traits [45]. It consists of 20 items each for anxiety state and traits that are scored on a 4-point scale. Higher scores indicate greater anxiety. High internal consistency of the STAI-S and test–retest reliability of the STAI-T have been reported for the Japanese version [46].

##### Symptom Checklist (SCL)-90R

This is a self-report questionnaire that assesses a broad range of psychological problems and symptoms of psychopathology including dimensions of somatization, obsessive-compulsive behaviors, interpersonal sensitivity, depression, anxiety, hostility, phobic anxiety, paranoid ideation, and psychoticism [47]. It consists of 90 items that are scored on a 5-point scale ranging from 0 to 4. Respondents are asked to indicate the status of psychological symptoms within the previous 7 days. Increased scores indicate more psychological symptoms. The Japanese version is currently under validation [48].

#### Possible predictors or moderators

##### General Functioning Subscale of the Family Assessment Device (GF-FAD)

This is a self-report questionnaire to assess family functioning based on the McMaster Model [49]. The general functioning subscale of the FAD (GF-FAD) consists of 12 items scored from 1 to 4. A high score indicates that the respondent considers the family functioning to be poor. It is commonly used in the field of mental illness [50] and is validated in Japanese [51].

##### Visual analog scale (VAS) of treatment expectations and satisfaction

Treatment expectation and satisfaction will be measured with a VAS. Each scale will consist of a horizontal 100 mm line with descriptors at the beginning and end to indicate the extremes of satisfaction, i.e., no satisfaction/totally dissatisfied and extreme satisfaction. Patients will rate their expectation and satisfaction by making a vertical mark along the length of the line. The measurement in millimeters will be converted to the same number of points ranging from 0 to 100. The question for expectation is “How much do you expect that the treatment will help you/how likely is it that the treatment will help you.” The question for satisfaction is “How satisfied are you with your treatment so far?”

### Sample size calculation

A sample size of 140 with 70 per group is estimated be sufficient to test the hypothesis based on previously published BN remission rates of 45% for CBT-E and 25% for TAU [29]. A sample size of 66 patients per treatment group is required to detect a difference of this magnitude with an 80% power for the primary analysis. The planned sample size was conservatively set to 70 subjects per group in consideration of differences in the methods of missing data handling from a previous study.

### Randomization

Randomization will be centrally performed with an electronic data capture system (HOPE eACReSS, Fujitsu ltd.). Randomization will be stratified by study center and BN severity (mild/moderate or severe/extreme) following the DSM-5 criteria.

### Statistical analysis

Outcome will be examined using an intention to treat analysis. All enrolled patients who were allocated to study groups will be included in the analysis of the data. Patients will be followed up for possible assessment even after withdrawal from treatment or from the study. Fisher’s exact test will be used for analysis of the primary outcome. For the primary outcome analysis, missing values will be treated as “not in remission of BN”. A per-protocol analysis including patients who complete at least 70% of the scheduled sessions will also be performed. Patients who received prohibited therapies (e.g., medication change or other structured psychosocial treatment) will be excluded from the analysis. For secondary outcomes, Fisher’s exact test will be applied to the binary variables, and mixed models for repeated measures will be used with continuous variables. The statistical analysis will be conducted independently by a statistician (KM) using SAS ver. 9.4 (https://www.sas.com/en_us/software/sas9.html).

### Dissemination

The study protocol has been registered in the clinical trial registration system at the University Hospital Medical Information Network Research Center (UMIN test ID: UMIN000031625; http://www.umin.ac.jp/ctr/index-j.htm). The results will be disclosed at the UMIN-CTR.

### Data management and monitoring

Study data will be entered immediately into the electronic data capture system at each participating center. On-site or off-site monitoring will be conducted. A Data and Safety Monitoring Board, who are independent from the study, will oversee the trial data and ethics.

### Ethical compliance

This protocol has been reviewed and approved by the Institutional Review Board of the (Japanese) NCNP (A2017–067, accepted on November 29, 2017) and the Ethics Committees at each of the study centers. Written informed consent will be obtained from all study participants. Although no harm is expected, monitoring for serious adverse events will be done. All data will be properly managed.

## Discussion

This will be the first RCT to investigate the effectiveness of ED-focused CBT treatment in Asia. The significance of this study lies in determining whether a specific ED-focused CBT, which is known to be effective in Western countries, is also effective in countries with different cultures and medical systems. Cultural differences may not be thought a priori to greatly influence the likely effectiveness of CBT-E because the ED-specific psychopathology is similar throughout cultures. However, there is currently no empirical support for CBT-E in Japan. The study will also provide valuable data comparing the outcomes achieved with ED-focused CBT with those achieved with the treatment currently offered to patients in Japan. The results will provide helpful information for service planning and training needs.

CBT-E was chosen because it has been extensively studied and supported [28]. In addition, the transdiagnostic nature of CBT-E [20] will enable extending its application to other EDs in Japan. Some previous studies on the effectiveness of CBT-E targeted all types of not-underweight patients with ED (i.e., a group of patients with BN, binge-eating disorders, and other unspecified eating disorders) [22, 24, 52]. In these transdiagnostic studies, the EDE or EDE-Q global score, with its associated population norms, has been used as an operational definition of remission (e.g., a global score of < 1 standard deviation above the community mean) [22, 24, 52]. Because neither the population norm of EDE nor EDE-Q scores are available in Japanese, it is difficult to make a unified, consensus definition of remission for a group of patients with various ED diagnoses. Therefore, the current study exclusively focused on BN patients rather than all non-underweight patients with EDs.

The study strengths include the RCT design and blinded assessment of treatment outcome. Patients will be followed up for 60 weeks after the end of treatment to determine whether treatment effects are maintained over the long-term. Multicenter recruitment makes it possible to enroll a large enough number of participants to ensure that the study is adequately powered. Single center RCTs have been reported to be prone to bias and tend to show larger intervention effects than those obtained by multicenter RCTs [53], whereas findings from multicenter RCTs tend to be robust and generalizable to real-world settings. Using TAU as a comparison group allows evaluation of the effects of CBT-E versus the best currently available treatment in Japan [30]. Both the CBT-E and TAU therapists will be experienced in the treatment of EDs. CBT-E will be implemented by competent therapists and its proper administration will be ensured. Our study has some limitations. One is associated with psychotherapy interventions in general. Because patients cannot be blinded to their treatment condition, the outcome may be affected by patient expectations concerning the two treatment conditions. This possibility is mitigated by measuring the effect of patient expectation and examining it in relation to outcome. The other limitation is a self-selection bias because participants will be recruited from the community using advertisements to ensure a sufficient number of samples.

## Data Availability

Not applicable because this is a protocol.

## Abbreviations

AN: Anorexia nervosa
BDI: Beck Depression Inventory
BN: Bulimia nervosa
CBT: Cognitive behavior therapy
CBT-E: Enhanced cognitive behavior therapy
CIA: Clinical Impairment Assessment Questionnaire
DSM: Diagnostic and Statistical Manual of Mental Disorders Fifth Edition
ED: Eating disorder
EDE: Eating Disorder Examination
EDE-Q: Eating Disorder Examination Questionnaire
GF-FED: General functioning subscale of the Family Assessment Device
MINI: Mini International Neuropsychiatric Interview
RCT: Randomized controlled trials
SCL-90R: Symptom Checklist-90R
STAI: State–Trait Anxiety Inventory
VAS: Visual analog scale
